# Evolution Mechanism of Sputtered Film Uniformity with the Erosion Groove Size: Integrated Simulation and Experiment

**DOI:** 10.3390/molecules28227660

**Published:** 2023-11-18

**Authors:** Guo Zhu, Yutong Yang, Baijun Xiao, Zhiyin Gan

**Affiliations:** 1 School of Mechanical & Electrical Engineering, Hunan City University, Yiyang 413000, China; yangyutong896@163.com (Y.Y.); xbjcai@sina.cn (B.X.); 2 School of Mechanical Science & Engineering, Huazhong University of Science & Technology, Wuhan 430074, China; zhiyingan@126.com

**Keywords:** Monte Carlo method, molecular dynamics, magnetron sputtering, sputtered particle transport, film thickness uniformity

## Abstract

In this work, Cu thin films were experimentally fabricated at different target–substrate distances by 2-inch and 4-inch circular planar magnetron targets. Meanwhile, the sputtering deposition of Cu thin films was investigated via an integrated multiscale simulation, where the magnetron sputtering discharge was modeled using the Monte Carlo (MC) method, and the sputtered particle transport was simulated using a coupled Monte Carlo (MC) and molecular dynamics (MD) method. Experimental results indicated that, as the target–substrate distance increased from 30 to 120 mm, the film thickness distribution of the 2-inch target sputtering changed from a bell-shaped curve to a line-shaped curve, while that of the 4-inch target sputtering varied from a saddle-shaped curve to a line-shaped curve. The simulation results were accordant with the experimental results. The simulation results revealed that, at a target–substrate distance of 30 mm, the sputtering particle flow from the 2-inch target overlapped strongly near the substrate center, leading to a bell-shaped film thickness distribution, while the increased diameter of the erosion groove on the 4-inch target reduced the superposition effect of the sputtering particle flow near the substrate center, resulting in a saddle-shaped film thickness distribution. In addition, when the target–substrate distance ranged from 30 to 120 mm, the film thickness uniformity of 4-inch target sputtering was superior to that of 2-inch target sputtering, and the underlying mechanism was discussed in detail.

## 1. Introduction

Due to the convenient process tuning and low coating temperature, magnetron sputtering is widely employed in manufacturing semiconductor, photo-electronic, and thermoelectric thin film devices [1,2,3]. The film thickness uniformity, as a critical index, significantly determines the performance consistency of film devices [4]. The thickness uniformity of the sputtered film is influenced by sputtering conditions, including the target–substrate geometrical configuration, relative motions between the target and substrate, and the target surface erosion. Investigating the interplay between sputtering conditions and film thickness uniformity is of great significance to improving the performance of thin film devices.

Currently, real-time monitoring for the sputtered film deposition cannot be realized through experimental means. Improvements in the thickness uniformity of sputtered films solely via empirical inference and posterior experiments are difficult, time-consuming, and expensive [5]. Consequently, the analytical models for the deposition uniformity of the circular planar single-target [6,7,8,9,10] and rectangular planar single-target [11,12] sputtering systems were successively developed to reinforce the understanding of experimental results and reveal the underlying mechanism. Then, extended analytical models were further proposed for the circular planar dual-target [13] and circular planar triple-target [14] sputtering systems. During magnetron sputtering deposition, fast-moving particles undergo no collision before they reach the substrate surface, while slow-moving particles are transported to the substrate surface by diffusion due to scattering collisions. However, the diffusion transport of slow-moving particles is neglected in the analytical model [14], although it significantly influences the film thickness distribution.

On the other hand, the sputtered particle transport can also be simulated by the MC method, in which the free flight and scattering of sputtered particles are both taken into consideration. Thus, a more precise distribution of deposited particles on the substrate can be obtained through MC simulation. In most MC simulations, the post-collision flight angles of colliding particles are approximately calculated based on the assumption that the argon atom is at rest before the collision [15,16,17,18,19,20,21,22], since the accurate calculation of the scattering angle requires complex coordinate transformations [23]. In this context, a coupled MC-MD method was developed to simulate the transport processes of sputtered particles [24], in which the velocities of colliding particles after collisions were calculated using the MD method. Furthermore, the initial emission positions of sputtered particles are determined by the ionization density distribution near the target surface during magnetron sputtering discharge [25]. However, due to the disconnect between the magnetron sputtering discharge research and sputtered particle transport research, the initial emission positions of sputtered particles in existing MC simulations are approximately selected based on the uniform distribution [16], Gauss distribution [17,26], or the measured surface profile of the etched target [27]. Accordingly, a combined numerical investigation of the magnetron sputtering discharge and sputtered particle transport is needed to precisely characterize the non-uniform erosion of the target surface, which is critical to understanding the control mechanism of sputtered film uniformity.

In the present work, a combined numerical investigation of the magnetron sputtering discharge and sputtered particle transport was conducted. In this combined numerical simulation, the magnetron sputtering discharge was modeled using the MC method, in which the sputtering possibility distribution on the copper target surface was evaluated based on the calculated ionization density distribution near the target surface. Then, the transport processes of sputtered Cu atoms were further simulated by the coupled MC-MD method, in which the initial emission positions of sputtered Cu atoms were selected from the calculated sputtering possibility distribution. In particular, the thickness distributions of sputtered films deposited from 2-inch and 4-inch targets were investigated through simulation and experiments, respectively. The evolution mechanism of sputtered film uniformity with the erosion groove size was skillfully explored by investigating the deposition behavior of sputtered atoms ejected from fan-shaped sputtering sources. This work attempted to bridge the gap between magnetron sputtering discharge research and sputtered atom transport research in the DC magnetron sputtering field, such that the state data of sputtered atoms arriving at the substrate surface can be calculated more realistically.

## 2. Results

### 2.1. The Radial Distributions of the Sputtering Possibility on the 2-Inch and 4-Inch Target Surface

**Figure 1:**
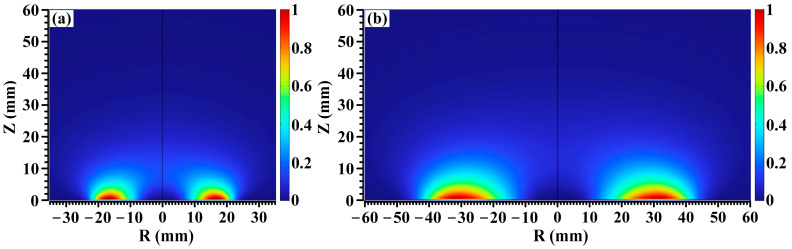
Distribution nephograms of ionization density for (**a**) 2-inch sputtering and (**b**) 4-inch target sputtering.

Figure 1 shows distribution nephograms of ionization density for the 2-inch target sputtering and 4-inch target sputtering, respectively. In Figure 1a,b, all the ionization density values were normalized by the maximum ionization density value in the simulation domain, and all the points in the simulation domain were colored based on the normalized ionization density values. Figure 1 displays the typical ionization density distribution nephograms of balanced magnetron sputtering discharge, which resembles those reported in ref. [28]. As shown in Figure 1a,b, ionization points are concentrated in the region approaching the target surface, since almost all the energetic electrons are trapped in this region with the magnetic field almost parallel to the target surface [29]. As the target diameter increases from 2 inches to 4 inches, the width and central diameter of the annular ionization regime both increase due to the increase in the width of the magnetic field on the target surface.

Argon ions, generated by the collision between energetic electrons and argon atoms, are accelerated by the electrostatic field between ionization points and the target surface. They almost impact perpendicularly onto the target surface, since the effect of the magnetic field on argon ion trajectory is negligible. The bombardment of argon ions results in the sputtering of near-surface target atoms. The sputtering yield is dependent on the bombarding energy. Herein, the sputtering density is introduced and defined as the number of sputtered atoms per unit area, which can be expressed by [30]

(1) $$S_{j}=\frac{\sum_{i=1}^{n_{0}}Y\left(E_{i}\right)}{2\pi r_{j}\Delta r}$$

where *Y* is the energy-dependent sputtering yield, *E*_i_ is the bombarding energy of the *i*-th argon ion, *n*_0_ is the total number of argon ions impinging the annular region with a radial coordinate of *r_j_* on the target surface, ∆*r* is the radial width of the annular region, and *S*_j_ is the sputtering density within the annular region. Based on Equation (1), the radial distributions of the sputtering density for the 2-inch and 4-inch targets can be calculated, respectively. Then, the sputtering possibility of a sputtered atom ejected from the *j*-th annular region can be calculated by

(2) $$P_{j}=\frac{S_{j}}{\sum_{1}^{n_{1}}S_{k}}$$

where *n*_1_ is the total number of the annular regions divided on the target surface. The radial distributions of the sputtering possibility on the target surface can be evaluated according to Equation (2). 

**Figure 2:**
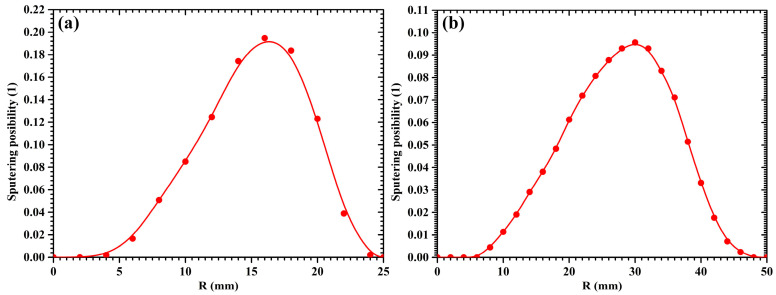
The radial distribution of sputtering possibility for (**a**) 2-inch target and (**b**) 4-inch target.

Figure 2 displays the radial distribution of the sputtering possibility for the 2-inch and 4-inch targets, respectively. As shown in Figure 2, the peaks of the sputtering possibility distribution are situated at the radial coordinates of ±16 mm for the 2-inch target sputtering, while the peaks of the sputtering possibility distribution are located at the radial coordinates of ±30 mm for the 4-inch target sputtering. These radial coordinates correspond to the points on the target surface where the vertical component of the magnetic field is zero [31]. Therefore, the initial emission position (*r_j_*) of a sputtered atom can be sampled from the sputtering possibility distribution via the acceptance–rejection method. Then, the *x* and *y* coordinates of the sputtered atom on the target surface can be determined by the random number *ϕ* distributed uniformly in [0, 2π].

*x* = *r_j_*cos(*ϕ*) (3)

*y* = *r_j_*sin(*ϕ*) (4)

### 2.2. Deposition Density Distribution of the 2-Inch Target Sputtering

**Figure 3:**
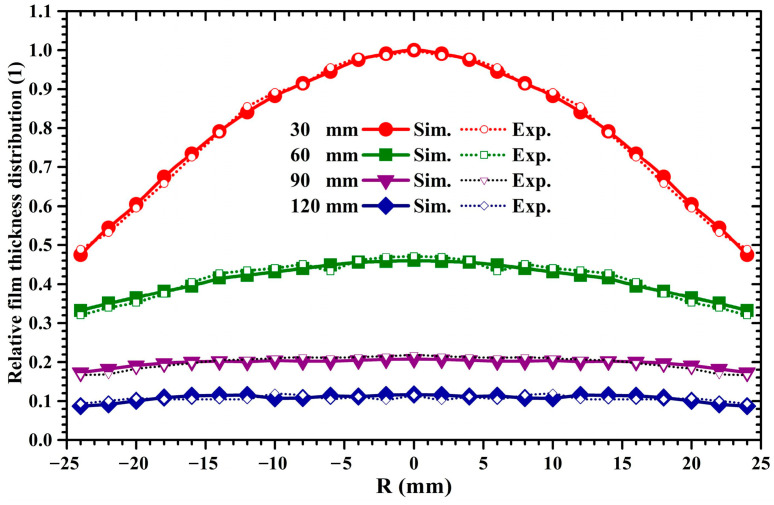
Relative film thickness distribution of Cu atoms sputtered from the 2-inch target under different target-substrate distances.

Figure 3 displays the variation in the thickness distribution of the sputtered film deposited from the 2-inch target with the target–substrate distance. In Figure 3, the experimentally measured film thickness distributions are depicted by dotted curves, while the calculated deposition density distributions are graphed by colored solid curves. Herein, the deposition density is defined as the number of deposited Cu atoms on the substrate per unit area. To evaluate the radial deposition density distribution on the substrate in the simulation, the circular substrate plane was partitioned into 13 regions, including 1 circular region and 12 concentric annular regions. The radius of the circular region and the radial width of the annular regions were set to 1 and 2 mm, respectively. In each simulation, the number of Cu atoms deposited in the *j*-th region was recorded and then divided by the area of the *j*-th region. Accordingly, the relationship between the film thickness and deposition density can be expressed as follows:

(5) $$T_{j}=\frac{mn_{j}}{{\rho A}_{j}}=k\frac{n_{j}}{A_{j}}={kD}_{j}$$

where *T_j_* is the film thickness in the *j*-th region, *m* is the mass of Cu atom, *n*_j_ is the number of Cu atoms deposited in the *j*-th region, *ρ* is the coating density, *A_j_* is the area of the *j*-th region, *k* is the ratio of *m* and *ρ*, and *D_j_* is the deposition density. Therefore, the film thickness is proportional to the deposition rate. To intuitively represent the variation in the film thickness uniformity, all the calculated deposition density values within the 13 regions at various target–substrate distances were normalized by that in the circular region when the target–substrate distance was set to 30 mm. Accordingly, Figure 3 represents the relative film thickness distributions for the 2-inch target sputtering. To compare the experimentally measured results with the simulated results, the measured film thickness values at various target–substrate distances were normalized by that in the circular region when the target–substrate distance was set to 30 mm. It is clear that the calculated distributions are consistent with the experimentally measured distributions at all target–substrate distances. It can be seen from Figure 3 that, when the target–substrate distance is 30 mm, the film thickness distribution has a bell-shaped profile, where the film thickness at the substrate center is much greater than that near the substrate margin. With the increase in the target–substrate distance from 30 to 120 mm, the bell-shaped film thickness distribution gradually varies to an arch-shaped one and, ultimately, to a line-shaped one. It can be found that the increase in the target–substrate distance is beneficial for improvements in film thickness uniformity but at the expense of the deposition rate.

### 2.3. Deposition Density Distribution of the 4-Inch Target Sputtering

**Figure 4:**
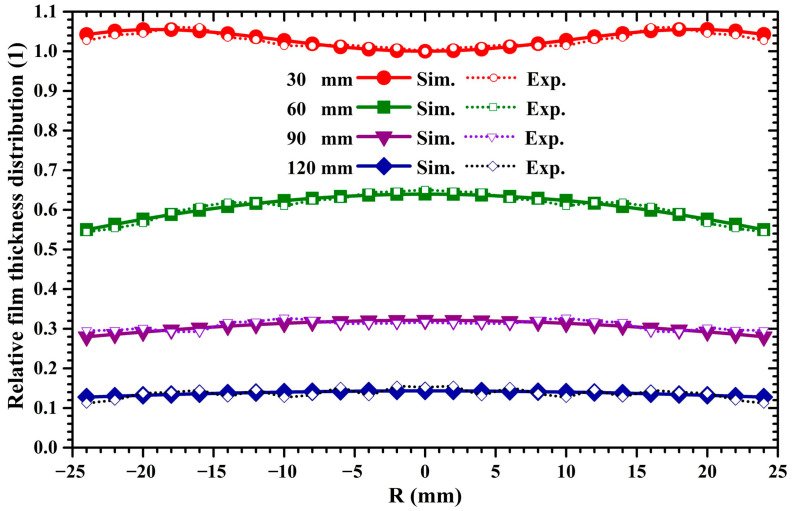
Relative film thickness distribution of Cu atoms sputtered from the 4-inch target under different target–substrate distances.

Figure 4 displays the variation in the thickness distributions of sputtered Cu films deposited from the 4-inch target with the target–substrate distance. In Figure 4, the experimentally measured film thickness distributions are depicted by colored dotted curves, while the calculated deposition density distributions are graphed by colored solid curves. To compare the experimentally measured results with the simulated results, the measured film thickness values and calculated deposition density values were normalized by the same scheme mentioned in Section 2.2. Accordingly, Figure 4 plots the relative film thickness distribution. It is clear that the calculated distributions are accordant with the experimentally measured distributions at all target–substrate distances. It can be found from Figure 4 that, when the target–substrate distance is 30 mm, the distribution profile of the film thickness is a saddle-shaped curve, where the film thickness at the substrate center is lower than that near the substrate margin. With the increase in the target–substrate distance from 30 to 120 mm, the saddle-shaped film thickness distribution gradually transforms to an arch-shaped one and, further, to a line-shaped one. It also can be seen that the increase in the target–substrate distance leads to a reduction in the deposition rate. 

### 2.4. Film Thickness Uniformity of 2-Inch and 4-Inch Target Sputtering

**Figure 5:**
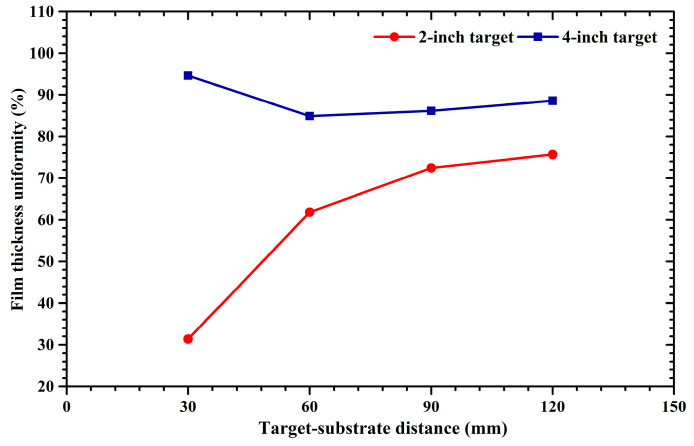
Thickness uniformity of sputtered films deposited on 2-inch substrate for 2-inch and 4-inch target sputtering under different target–substrate distances.

The thickness uniformity of a sputtered film (U) can be evaluated using the following equation [32]:

(6) $$U=100\%\;-\frac{2{(H}_{\max}-H_{\min})}{H_{\max}+H_{\min}}\times 100\%$$

where the *H*_max_ and *H*_min_ represent the maximum and minimum film thickness, respectively. Figure 5 displays the thickness uniformity of sputtered films deposited by 2-inch and 4-inch targets under different target–substrate distances. In Figure 5, variation curves of film thickness uniformity for 2-inch and 4-inch target sputtering are depicted by red and blue lines, respectively. It can be seen that, as the target distance increases from 30 to 120 mm, the film thickness uniformity of 2-inch target sputtering increases monotonically, while the film thickness uniformity of 4-inch target sputtering decreases first and then increases. The film thickness uniformity of 4-inch target sputtering is superior to that of 2-inch target sputtering. As the target–substrate distance increases from 60 to 120 mm, the film thickness uniformity of 2-inch and 4-inch target sputtering gradually increases. It is known that sputtered atoms have an anisotropic initial emission angular distribution [33]. As the sputtering pressure is 0.5 Pa, the mean free paths of sputtered atoms with 2 eV and 30 eV kinetic energy are 41.6 mm and 113.7 mm, respectively, and the percentage of these sputtered Cu atoms is around 70% [24]. This suggests that, at a target–substrate distance of 30 mm, most of the sputtered atoms undergo no collision before they arrive at the substrate surface. Accordingly, the anisotropic ejection of sputtered atoms results in the bell-shaped and saddle-shaped film thickness profiles shown in Figure 3 and Figure 4 under a target–substrate distance of 30 mm. When the target–substrate distance is set to 120 mm, it exceeds the mean free path of sputtered atoms possessing energy of 30 eV. Thus, most of the sputtered atoms will experience scattering collisions before they arrive at the substrate surface. The flying angles of sputtered atoms gradually vary due to the increase in the scattering collision frequency with the target–substrate distance, leading to a quasi-isotropic incident angular distribution of sputtered atoms when they approach the substrate surface [15,34]. Accordingly, since the anisotropy of the incident angular distribution gradually decreases with the target–substrate distance, the arch-shaped or saddle-shaped film thickness distribution is gradually transformed into the line-shaped one shown in Figure 3 and Figure 4, resulting in an improvement in the film thickness uniformity.

## 3. Discussion

More interestingly, for the 2-inch target sputtering, sputtered Cu atoms were initially emitted from the annular erosion region of the target surface in our simulation. However, under a target–substrate distance of 30 mm, the film thickness distribution had a bell-shaped profile, in which the maximum value of the film thickness appeared at the substrate center (*R* = 0 mm). As the target diameter increased from 2 to 4 inches, this bell-shaped film thickness distribution was varied to a saddle-shaped one, leading to an improvement in the film thickness uniformity. To understand the underlying mechanism, the deposition behavior of Cu atoms sputtered from fan-shaped sputtering sources on 2-inch and 4-inch targets was further studied via supplemental simulations, respectively. 

**Figure 6:**
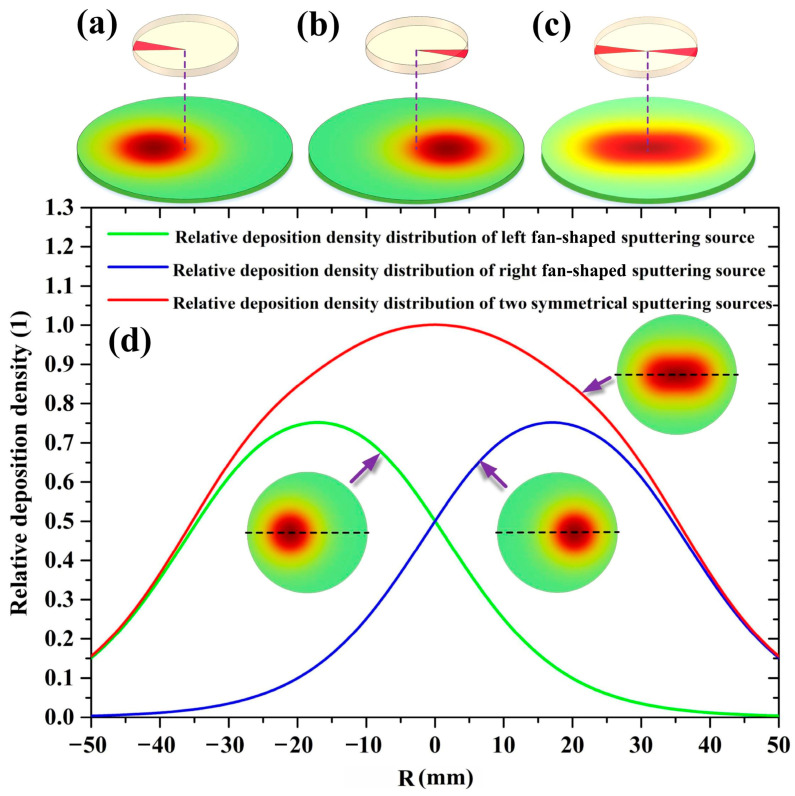
The deposition density distribution nephograms of (**a**) a left fan-shaped sputtering source, (**b**) a right fan-shaped sputtering source, and (**c**) two symmetrical fan-shaped sputtering sources on the 2-inch target, and (**d**) the radial deposition density distribution profiles of the three kinds of sputtering sources.

Figure 6a–c show the deposition density distribution nephograms of a left fan-shaped sputtering source, a right fan-shaped sputtering source, and two symmetrical fan-shaped sputtering sources on the 2-inch target surface under a target–substrate distance of 30 mm. Furthermore, Figure 6d displays the radial deposition density distributions of the three kinds of sputtering sources, which are plotted by the green, blue, and red solid curves, respectively. In Figure 6d, all the deposition density values are normalized by the maximum value in the radial deposition density distribution of two symmetrical fan-shaped sputtering sources. For the sputtering of the left (right) fan-shaped source, the radial deposition density distribution is an arch-shaped curve, whose peak value appears at the projection on the substrate of the point with the maximum sputtering possibility in the fan-shaped source. It can be found that, as the number of fan-shaped sputtering sources increases from 1 to 2, the arch-shaped deposition density distribution is varied to a bell-shaped one, which is analogous to the red solid curve shown in Figure 3. This suggests that the sputtered particle flow from all infinitesimal fan-shaped sputtering sources of the erosion groove overlaps near the substrate center. This strong superposition effect near the substrate center accounts for the bell-shaped deposition density distribution of the 2-inch sputtering target at a short target–substrate distance.

**Figure 7:**
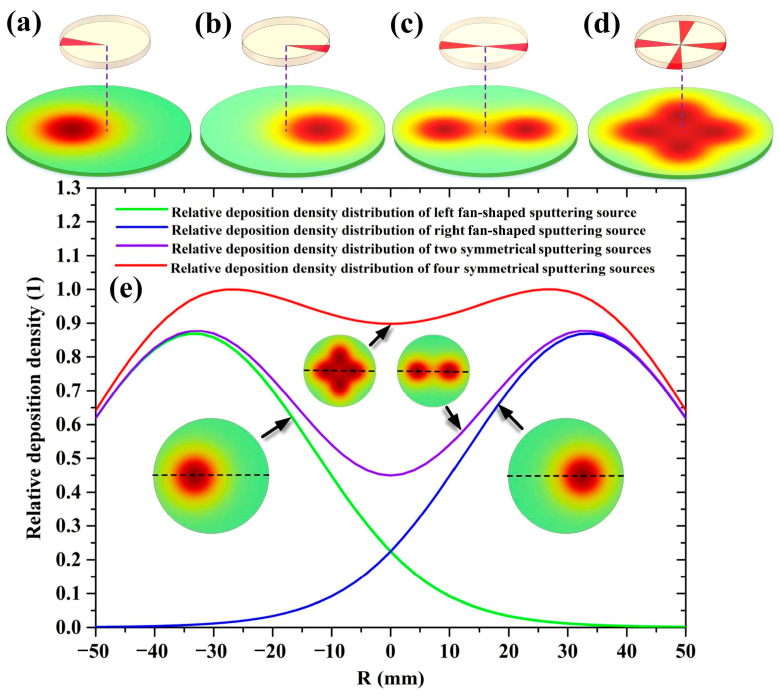
The deposition density distribution nephograms of (**a**) a left fan-shaped sputtering source, (**b**) a right fan-shaped sputtering source, (**c**) two symmetrical fan-shaped sputtering sources, and (**d**) four symmetrical fan-shaped sputtering sources on the 4-inch target, and (**e**) the radial deposition density distribution profiles of the four kinds of sputtering sources.

Figure 7a–d display the deposition density distribution nephograms of a left fan-shaped sputtering source, a right fan-shaped sputtering source, two symmetrical fan-shaped sputtering sources, and four symmetrical fan-shaped sputtering sources on the 4-inch target surface under a target–substrate distance of 30 mm. Figure 7e shows the radial deposition density distributions of the four kinds of sputtering sources, which are depicted by the green, blue, purple, and red solid curves, respectively. In Figure 7e, all the deposition density values are normalized by the maximum value in the radial deposition density distribution of four symmetrical fan-shaped sputtering sources. It can be seen that, as the number of fan-shaped sputtering sources increases from 1 to 4, the radial deposition density distribution gradually transforms from an arch-shaped curve to a saddle-shaped curve, which resembles the red solid curve shown in Figure 4. Accordingly, it can be concluded that, in the vicinity of the substrate center, the superposition of the arch-shaped deposition density distributions of infinitesimal fan-shaped sources results in the saddled-shape deposition density distribution of the 4-inch sputtering target when the target–substrate distance is set to 30 mm. Furthermore, comparing Figure 7d with Figure 7e, it can be found that, as the target diameter increases from 2 to 4 inches, both peak points of the arch-shaped deposition density distributions of the left and right fan-shaped sputtering sources move towards the substrate margin, which is mainly due to the increase in the diameter of the annular erosion groove. The enlargement of the spacing distance between these peak points weakens the superposition effect of deposition density near the substrate center. Consequently, deposition density values near the substrate center are less than those near the peak points of the deposition density distributions of left and right fan-shaped sputtering sources, leading to a saddle-shaped deposition density distribution of the 4-inch sputtering target at a short target–substrate distance.

**Figure 8:**
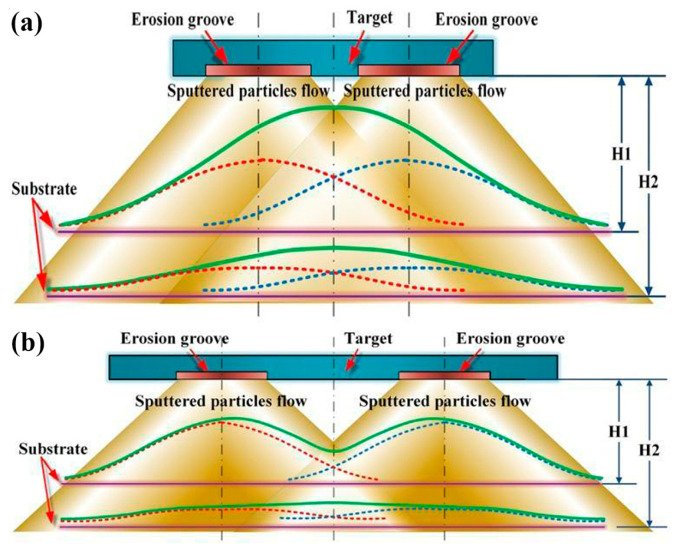
The influence mechanism of the diameter of the erosion groove on the film thickness distribution and the variation of the film thickness distribution with the target–substrate distance for (**a**) 2-inch target sputtering and (**b**) 4-inch target sputtering.

Figure 8 presents a schematic diagram to explain the underlying mechanism for the variation in film thickness distribution with the diameter of the erosion groove. Given the central symmetry of the spatial distribution of sputter particle flow, for simplification, Figure 8 only displays the superposition of sputtered particle streams on a cross-section. In Figure 8, the deposition density distributions of left and right infinitesimal sputtering sources are plotted with red and blue dash curves, respectively, while the entire deposited density distributions on the substrate are depicted with green solid curves. The anisotropic ejection of sputtered atoms eventuates an arch-shaped deposition density distribution of an infinitesimal sputtering source. The increase in the scattering collision frequency with the target–substrate distance leads to a quasi-isotropic flying angle distribution of sputtered atoms. Consequently, the uniformity of the entire film thickness distribution is gradually improved. In addition, since the possibility of sputtered atoms moving outside the substrate region increases with the collision frequency, the film deposition rate gradually decreases with the target–substrate distance. For the sputtering of the target with a small diameter, in the vicinity of the substrate center, the strong superposition of the sputtering particle flow from infinitesimal sputtering sources results in a bell-shaped entire film thickness distribution at a short target–substrate distance. This bell-shaped film thickness distribution was also reported in ref. [35], in which the diameter of the sputtering target was 50 mm. With the increase in the target–substrate distance, the superposition of more uniform deposition density distributions of infinitesimal sputtering sources gives rise to an increasingly flat entire film thickness distribution on the substrate, which is consistent with the variations shown in Figure 3. Furthermore, as the diameter of the target increases, the entire film thickness distribution might have a saddle-shaped profile at a short target–substrate distance due to the reduction in the superposition effect of sputtering particle flow near the substrate center. With the increase in the target–substrate distance, the film thickness distribution becomes comparatively uniform owing to the enchantment in the scattering effect, which coincides with the variations shown in Figure 4. The variation in the film thickness profile with the target–substrate distance shown in Figure 8b was also reported in Refs. [7,36].

## 4. Materials and Methods

### 4.1. Experiment of Cu Film Deposition and Film Thickness Measurement

In the experiment, two copper targets with a purity of 99.99% were used as sputtering sources, whose diameters were 50.8 and 101.6 mm, respectively. The (001) silicon wafer with a diameter of 50.8 mm was chosen as the substrate. The substrate was placed in acetone and cleaned via ultrasonication for 3 min. Then, it was washed using deionized water. A multifunctional magnetron sputtering system, including 2-inch and 4-inch balanced magnetron sputtering sources produced by Sky Technology Development Co., Ltd. (Hunnan, Shenyang, China), was employed to prepare sputtered Cu films. The target voltage was set to −400 V. The pressure of the background gas was maintained at 0.5 Pa. The deposition time of the sputtered film was set to 10 min. The target–substrate distance was varied from 30 mm to 120 mm.

**Figure 9:**
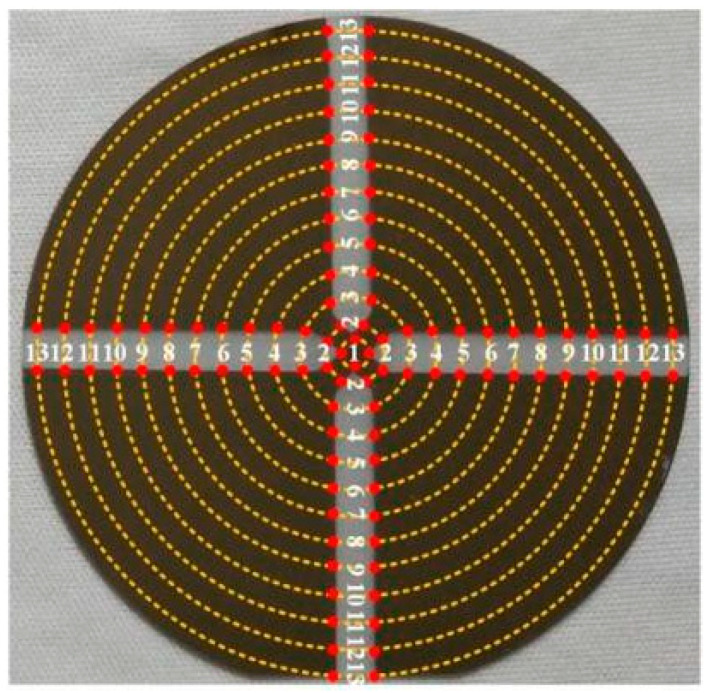
Measurement points on the eight steps in the deposited film. These points are marked by red dots and numbered from 1 to 13. The points located on an identical dashed circle are numbered by the same number.

Figure 9 shows the measurement scheme of the film thickness. Prior to the preparation of Cu film, four rotational symmetrically arranged rectangular regions on the substrate surface were masked by polyimide films with a width of 3 mm, such that eight steps in the sputtered Cu film can be formed. To qualify the Cu film thickness, the step height between the masked and unmasked region of the substrate was measured using a Bruker DektakXT surface profilometer (Billerica, MA, USA) [9,37]. Thirteen measurement points numbered from 1 to 13 were chosen on each step. Therefore, the eight measurement points marked by the same number i (1 ≤ i ≤ 13) on the eight steps were located on an identical circle with a radius of R_i_. Then, the mean value of the film thickness at these eight measurement points was calculated and recorded as the film thickness value at R_i_, such that the radial film thickness distribution could be obtained.

### 4.2. MC Simulation of Magnetron Sputtering Discharge

DC magnetron sputtering discharge was simulated using the MC method. In the MC simulation, the target and substrate of the magnetron sputtering system are in a parallel and coaxial configuration. The target voltage was set to −400 V. The pressure of the background gas was maintained at 0.5 Pa. Since the effect of gas heating can be neglected during the DC magnetron sputtering discharge under pressure of less than 1 Pa [38], the temperature of the background gas was set to 300 K. 

In this MC simulation of the DC magnetron sputtering discharge, the magnetic and electric fields were assumed to be time-independent and location-dependent [39]. At the beginning of the simulation, it was supposed that electrons were uniformly ejected from the target surface with an initial energy of zero. Then, they underwent cycloidal spiral oscillation under the action of electrostatic field force and Lorentz force in the non-uniform electromagnetic field. Their motion equation can be expressed by

(7) $$\frac{dv}{dt}=\frac{q}{m}\left(E+v\times B\right)$$

where *E* and *B* represent the intensity of the electric field and magnetic field, respectively, and *v*, *m*, and *q* denote the velocity, mass, and charge of the electron, respectively. It was postulated that argon gas was in a thermal equilibrium state. Argon ions were generated by the collisions between argon atoms and energetic electrons. In the present simulation, elastic scattering, excitation, and ionization collisions were taken into consideration, whose occurrences were determined by the corresponding momentum transfer cross-sections [40]. The energy loss of an electron in the excitation and ionization collisions was set to 11.6 eV and 15.8 eV [30], respectively. The movement of primary and secondary electrons was traced during the simulation, and the specific simulation procedure was previously described in ref. [41]. In particular, the electric potential between the target and substrate was assumed to obey the distribution introduced in ref. [41]. A Hall sensor was adopted to measure the horizontal and vertical components of the magnetron field [42]. During the movement of the electron, if an ionization collision between the electron and argon atom occurred, the coordinate of the collision point was recorded and further used to calculate the ionization density distribution. The electron would be killed when it moved outside the simulation domain or its energy was lower than the ionization threshold energy of 15.8 eV.

### 4.3. MC-MD Simulation Method of Sputtered Particle Transport

The initial emission positions of sputtered atoms were determined by the sputtering possibility distribution on the target surface calculated based on the MC simulation results, and the specific procedures were introduced in Section 2.1. The initial emission energy of sputtered atoms was assumed to obey the Thompson distribution [43] and sampled via the rejection algorithm [15,44]. Yamamura’s angular distribution [33] was used to choose the initial emission polar angles of sputtered atoms. The emission azimuth angles of sputtered atoms, due to symmetry, were supposed to be distributed uniformly between 0 and 2π. After that, the transport processes of sputtered atoms could be modeled using the coupled MC-MD method, whose simulation procedures were introduced in our previous paper [24]. Ultimately, the deposition density distributions of 2-inch target sputtering and 4-inch target sputtering can be calculated, respectively.

## 5. Conclusions

In this work, 2-inch and 4-inch circular planar magnetron cooper targets were utilized to prepare Cu thin films at different target–substrate distances, respectively. Simultaneously, the MC method was used to simulate the magnetron sputtering discharge and calculate the ionization density distribution near the target surface. Then, the transport processes of sputtered atoms were modeled by a coupled MC-MD method based on the calculated results of the MC simulation. Experimental results suggested that, with the increase in the target–substrate distance, the film thickness uniformity was gradually improved, while the deposition rate gradually decreased. In particular, as the target–substrate distance increased from 30 to 120 mm, the bell-shaped film thickness distribution of the 2-inch target sputtering was gradually changed to a line-shaped one, while the saddle-shaped film thickness distribution of the 4-inch target sputtering was gradually varied to a line-shaped one. The simulation results agreed well the with experimental results. The MC simulation results suggested that the peak points of the V-shaped radial ionization density distributions of the 2-inch and 4-inch target sputtering were located at radial coordinates of 16 and 30 mm, respectively. Then, the radial sputtering possibility distributions of the 2-inch and 4-inch targets were obtained based on the calculated ionization density distributions. The MC-MD simulation results revealed that, when the target–substrate distance was set to 30 mm, the strong superposition of the sputtering particle flow near the substrate center led to a bell-shaped film thickness distribution of the 2-inch target sputtering, and the reduction in this superposition effect of the sputtering particle flow, resulting from the increased diameter of the erosion groove, resulted in a saddle-shaped film thickness distribution of the 4-inch target sputtering. In addition, the scattering collision frequency increased with the target–substrate distance, eventuating a quasi-isotropic incident angle distribution of deposited atoms and, thus, a comparatively uniform film thickness profile. This work proposed an integrated simulation scheme for the magnetron sputtering discharge and sputtered particle transport, which facilitates studying the dependence of the sputtered film uniformity on sputtering conditions.

## Data Availability

Data are contained within the article.
